# Author Correction: Parvalbumin neurons enhance temporal coding and reduce cortical noise in complex auditory scenes

**DOI:** 10.1038/s42003-025-08292-5

**Published:** 2025-06-25

**Authors:** Jian Carlo Nocon, Howard J. Gritton, Nicholas M. James, Rebecca A. Mount, Zhili Qu, Xue Han, Kamal Sen

**Affiliations:** 1 Neurophotonics Center, Boston University, Boston, 02215 MA USA; 2 Center for Systems Neuroscience, Boston University, Boston, 02215 MA USA; 3 Hearing Research Center, Boston University, Boston, 02215 MA USA; 4 Department of Biomedical Engineering, Boston University, Boston, 02215 MA USA; 5 Department of Comparative Biosciences, University of Illinois, Urbana, 61820 IL USA; 6 Department of Bioengineering, University of Illinois, Urbana, 61820 IL USA

**Keywords:** Neural circuits, Neural decoding, Cortex, Computational neuroscience

Correction to: *Communications Biology* 10.1038/s42003-023-05126-0, published online 19 July 2023

In the version of the article, there was an error in the code used to calculate discrimination performance where in the spike distance-based template matching algorithm used to calculate discrimination performance, a small fraction of template matches erroneously considered a spike train’s distance to itself, causing a small upward bias in discrimination performance of 1.2% on average. Following reanalysis, the majority of results (76/80) from statistical tests of significance did not change in significance, i.e., results that were statistically significant remained so, and results that were not significant (NS) remained so.

In a minority of cases (4/80) that were previously close to the level of significance (*p* < 0.05), the results of statistical tests changed from significant to not significant or vice versa. These cases include the following: 1, 2, reductions in spike count distance-based performance (Fig. 4c) is now significant for clean trials (*p* = 0.0590 → 0.0434) and no longer significant for masked trials (*p* = 0.020 → 0.0663); 3, reductions in van Rossum distance-based performance with a decay constant of 1 ms (Table 1) are no longer significant for masked trials (*p* = 0.0226 → 0.1438); 4, for supplemental analyses, the reduction in SPIKE-distance-based performance in multi-unit responses (Supplementary Fig. 7c) is no longer significant during clean trials (*p* = 0.040 → 0.0576). For each of these cases, the original text did not contain any specific statements regarding statistical significance, while original statements regarding spike counts, time scales and decreased performance remain accurate.

While the main text remains largely unchanged following reanalysis, numerical values have been corrected in the following figures and legends: Fig. 1d, Fig. 2d–f, all subplots in Figs. 3–6, Fig. 7b, and all subplots in Supplementary Figs. 7 and 8. For comparison, the original article and Supplementary Figs. are available alongside this amendment in the Supplementary information. The custom code used in the study has also been updated in the Code availability section and is now available at 10.5281/zenodo.14290575. All changes are now reflected in the HTML and PDF versions of the article.

## Supplementary information

Supplementary information

