# Supplementary material for: Parvalbumin neurons enhance temporal coding and reduce cortical noise in complex auditory scenes
Source: Commun Biol. 2023 Jul 19;6:751. doi: 10.1038/s42003-023-05126-0 (PMC10356822; doi:10.1038/s42003-023-05126-0)
Supplement: Supplementary file 2 — Supplementary Information [file 42003_2023_5126_MOESM2_ESM.pdf]

Parvalbumin neurons enhance temporal coding and reduce cortical noise in complex auditory scenes

Authors: Jian Carlo Nocon[1,2,3,4], Howard J. Gritton[5,6], Nicholas M. James[1,2,3,4], Rebecca A. Mount[1,2,3,4], Zhili Qu[5,6], Xue Han[1,2,3,4], Kamal Sen[1,2,3,4,*]

[1]Neurophotonics Center, Boston University, Boston, Massachusetts, United States of America 02215,

[2]Center for Systems Neuroscience, Boston University, Boston, Massachusetts, United States of America, 02215

[3]Hearing Research Center, Boston University, Boston, Massachusetts, United States of America, 02215

[4]Department of Biomedical Engineering, Boston University, Boston, Massachusetts, United States of America, 02215

[5]Department of Comparative Biosciences, University of Illinois, Urbana, Illinois, United States of America, 61820

[6]Department of Bioengineering, University of Illinois, Urbana, Illinois, United States of America, 61820

[*] Corresponding Author: kamalsen@bu.edu

Classification: Biological Sciences/Neuroscience

Keywords: Parvalbumin, cortical code, temporal code, cortical noise, cocktail party problem, complex scene analysis

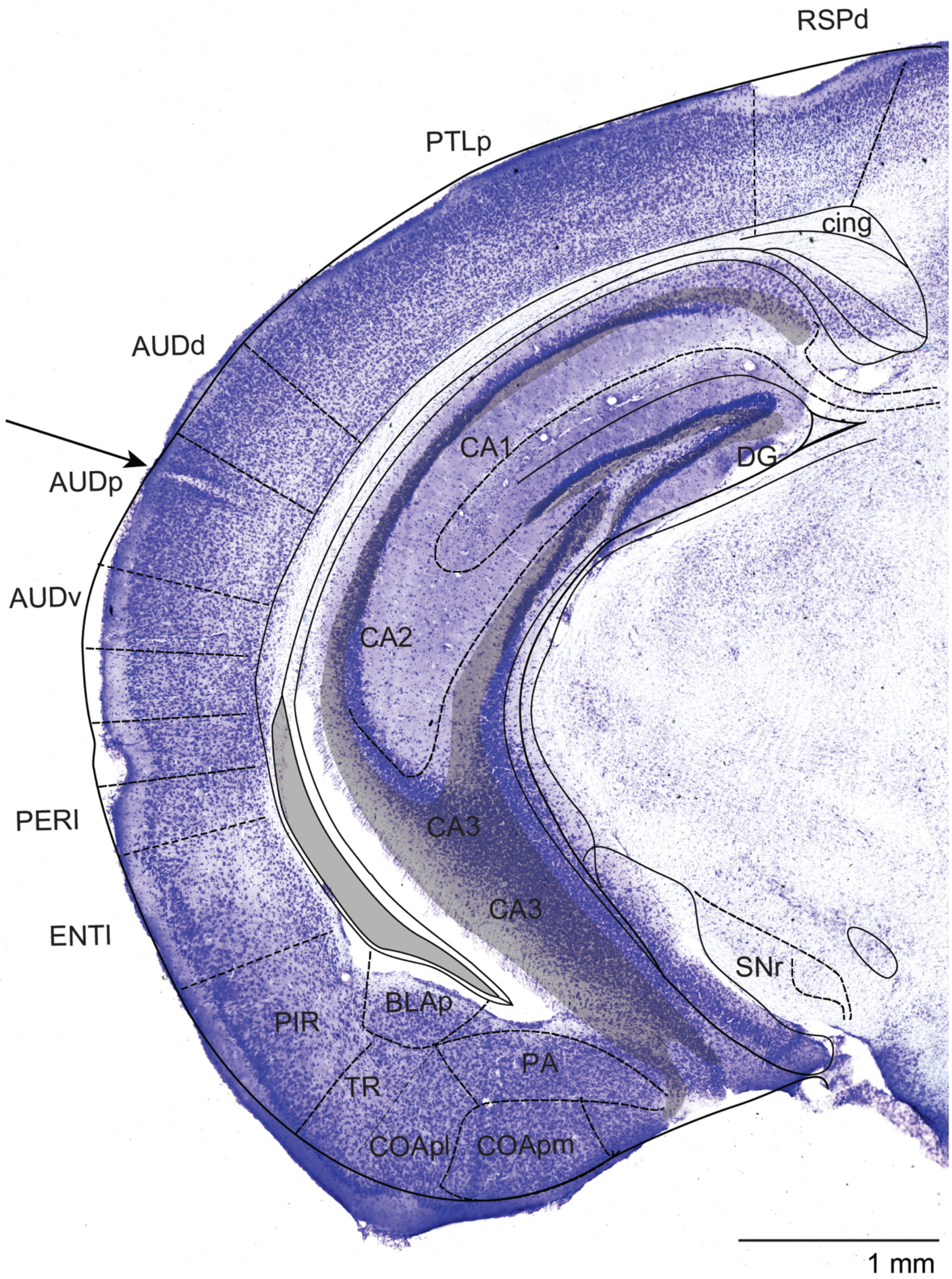

Supplementary Figure 1. Electrode placement in auditory cortex. Representative image from a nissl stained histological section showing electrode location. Arrow indicates location of electrode placement within coronal section that targets primary auditory cortex (AUDp). Image is overlayed with topography based on the Allen mouse brain atlas[1]. AUDd: dorsal auditory area; AUDp: primary auditory area; AUDv: ventral auditory area; BLAp: basolateral amygdalar nucleus, posterior part; CA1-3: fields CA1-3; cing: cingulum bundle; COApl: cortical amygdalar area, lateral part; COApm: cortical amygdalar area, medial part; DG: dentate gyrus; ENTl: entorhinal area, lateral part; PA: posterior amygdalar nucleus; PERI: perirhinal area; PIR: piriform area; PTLp: posterior parietal

association area; RSPd: retrosplenial area, dorsal part; SNr: substantia nigra, reticular part; TR: postpiriform transition area.

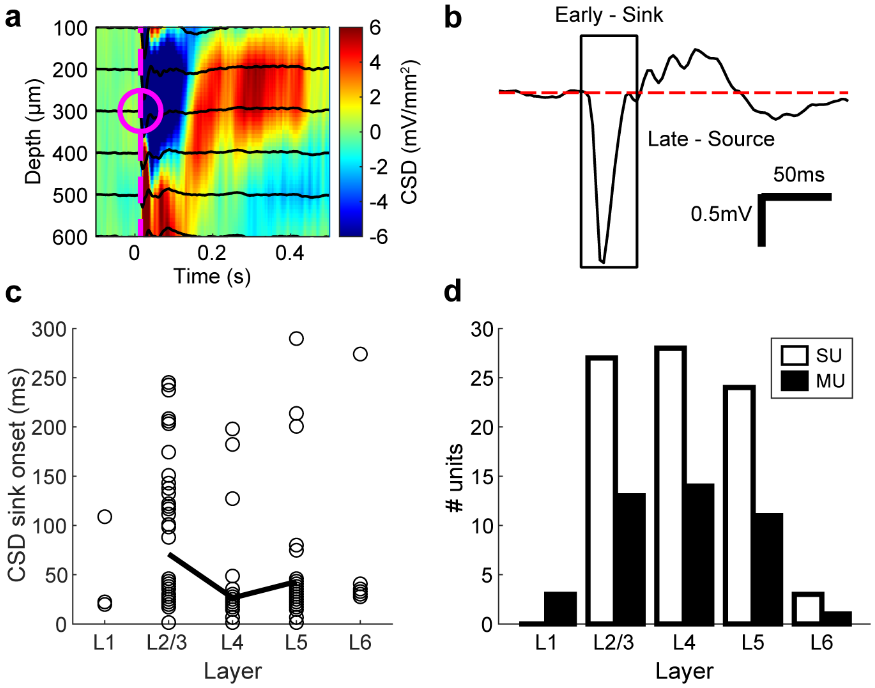

Supplementary Figure 2. Recording locations and density using CSD for layer separation. **a:** Current source density pseudo-colormap with event-related potential (ERP) traces overlaid in black. Magenta dashed line indicates time of earliest CSD sink onset, while the magenta circle indicates the channel with earliest onset. The mean CSD sink onset at all identified granular channels was 17.2 ms. **b:** Example ERP trace with CSD sink boxed in. Red dashed line indicates mean pre-stimulus activity, which was used to determine the threshold below which the CSD sink was detected. **c:** Layer vs. CSD sink onsets for all channels, with mean sink onset outlined in black from L2/3 to L5, with the mean CSD sink onset at L4 at 26.4ms. Mean sink onset for L1 and L6 are not shown due to the lack of units in both layers. **d:** Bar plot showing number of detected single units (SU, white, *n* = 82 units) and multi-units (MU, black, *n* = 42 units) at each layer.

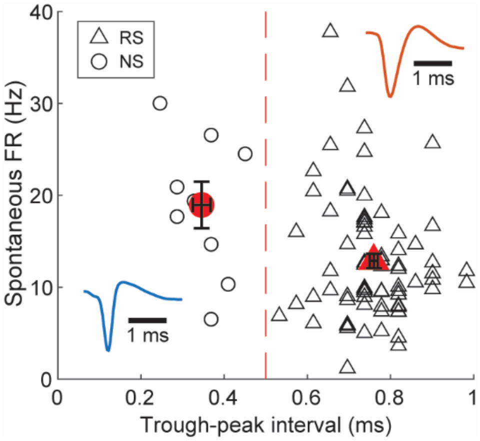

Supplementary Figure 3. Single units with narrow-spiking waveforms vs. regular-spiking waveforms. Scatter plot showing trough-peak interval of each single unit's spike waveform versus spontaneous firing rate during control trials. Dashed red line at 0.5ms represents the threshold between narrow-spiking units (NS, circle markers, *n* = 9 units) and regular-spiking units (RS, triangle markers, *n* = 73 units). Insets show example waveforms from a narrow-spiking single unit (bottom-left, blue) and regular-spiking single unit (top-right, orange) with scale bars measuring 1ms. Filled red markers represent the mean trough-peak interval and spontaneous firing rate for each unit type, with error bars representing ± SEM. Using two-sample t-tests with an assumption of unequal variance, both trough-peak interval (p = 3.92e-10, d = 4.42) and spontaneous firing rate (p = 0.0460, d = -0.89) were found to be significantly different between NS and RS units.

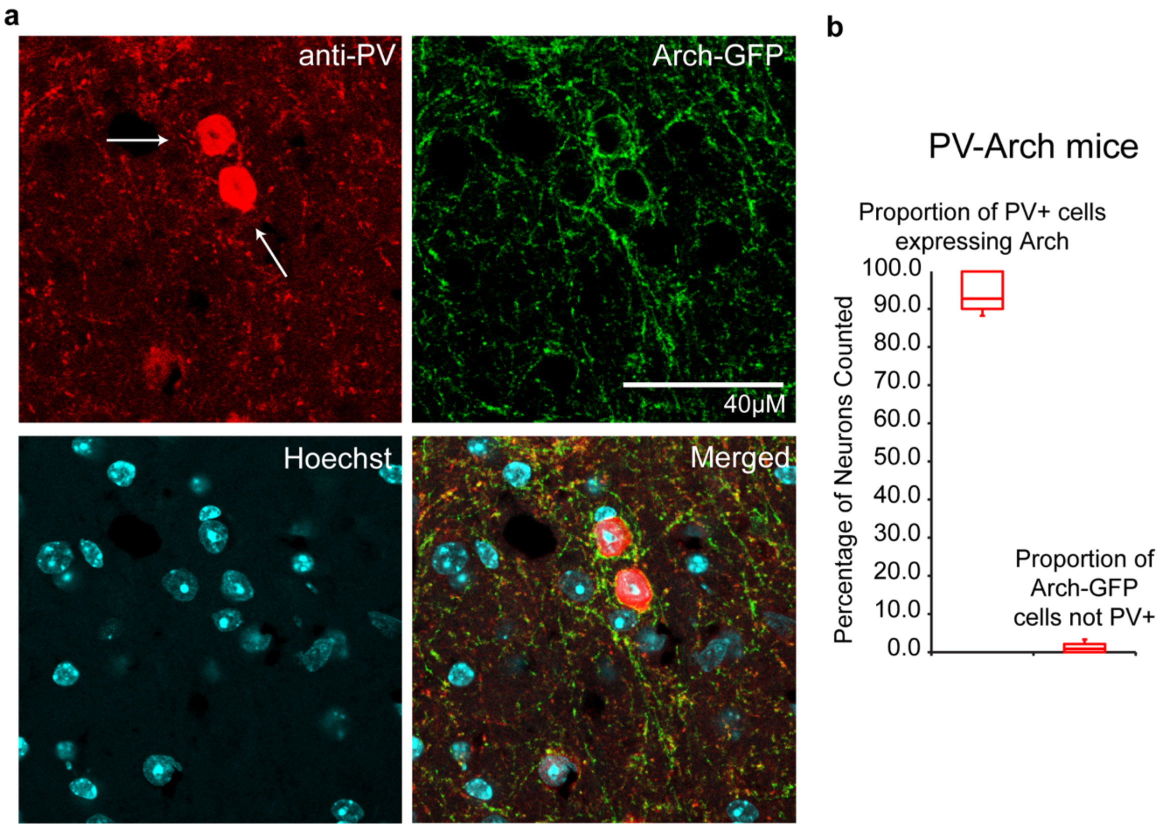

Supplementary Figure 4. Histology of PV-Arch mice: a: Post-study representative confocal photomicrograph example from PV-Arch mouse used in this study. Example includes merged images along with separate channel images for Arch-GFP (green), anti-PV immunofluorescence (red), and Hoechst labeling (cyan). White arrows indicate cells with co-localized expression of Arch and anti-PV. **b**: Box plot indicating quantification of viral specificity from auditory regions in PV-Arch mice recorded in this study ($n = 9$; see Methods in main text). 93.5% ± 1.0% (mean ± s.e.m.) of PV immunoreactive cells co-expressed Arch-GFP while 0.95% ± 0.23% (mean ± s.e.m.) of Arch-GFP cells were not immunoreactive for PV. For box plot figures, middle lines indicate the median, lower and upper lines of the box indicate quartiles below and above the median, and upper and lower whiskers indicate maximum or minimum values respectively.

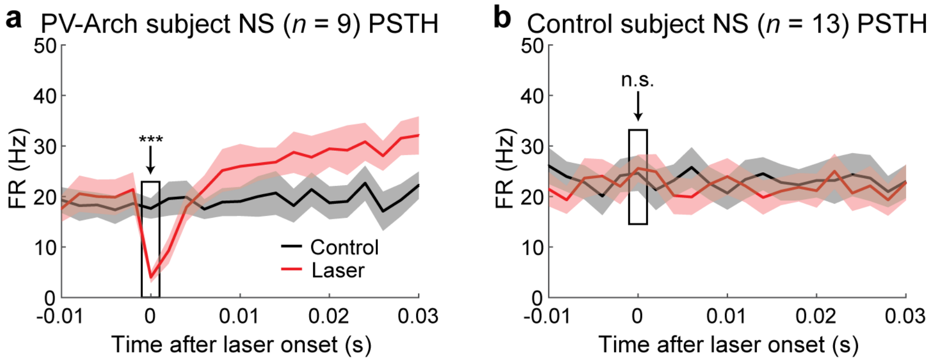

Supplementary Figure 5. NS units in PV-Arch subjects show immediate suppression following laser onset. Responses around laser onset for control (black) and optogenetic (red) conditions in n = 9 PV-Arch subjects **(a)** and n = 13 PV-only control subjects **(b)**. Since no auditory stimulus is present during this period, both clean and masked trials were used to construct PSTHs with bin size 2 ms. Shaded regions represent ± s.e.m. across subject responses. **a:** For PV-Arch subjects, a 2-sample t-test found that the mean spiking rate during the first 2 ms of optogenetic suppression was significantly lower (p = 2.30e-05, d = 2.77) than that of the control condition. Individually, 8 of the 9 NS PV-Arch units showed a significant decrease in spiking within the first 2 ms of optogenetic suppression compared to the control. **b:** For control subjects, a 2-sample t-test found that the mean spiking rate during the first 2 ms of laser onset was not significantly different between conditions (p = 0.830, d = -0.09). Individually, 12 of the 13 NS PV only units did not show a significant change in spiking during the first 2 ms of laser stimulation compared to control. In addition, we compared the change in spiking during laser onset (control minus laser) between Arch-expressing and non-Arch-expressing subjects. An un-paired, 2-sample t-test yielded a significant difference between the two groups (p = 8.42e-05, d = 2.13).

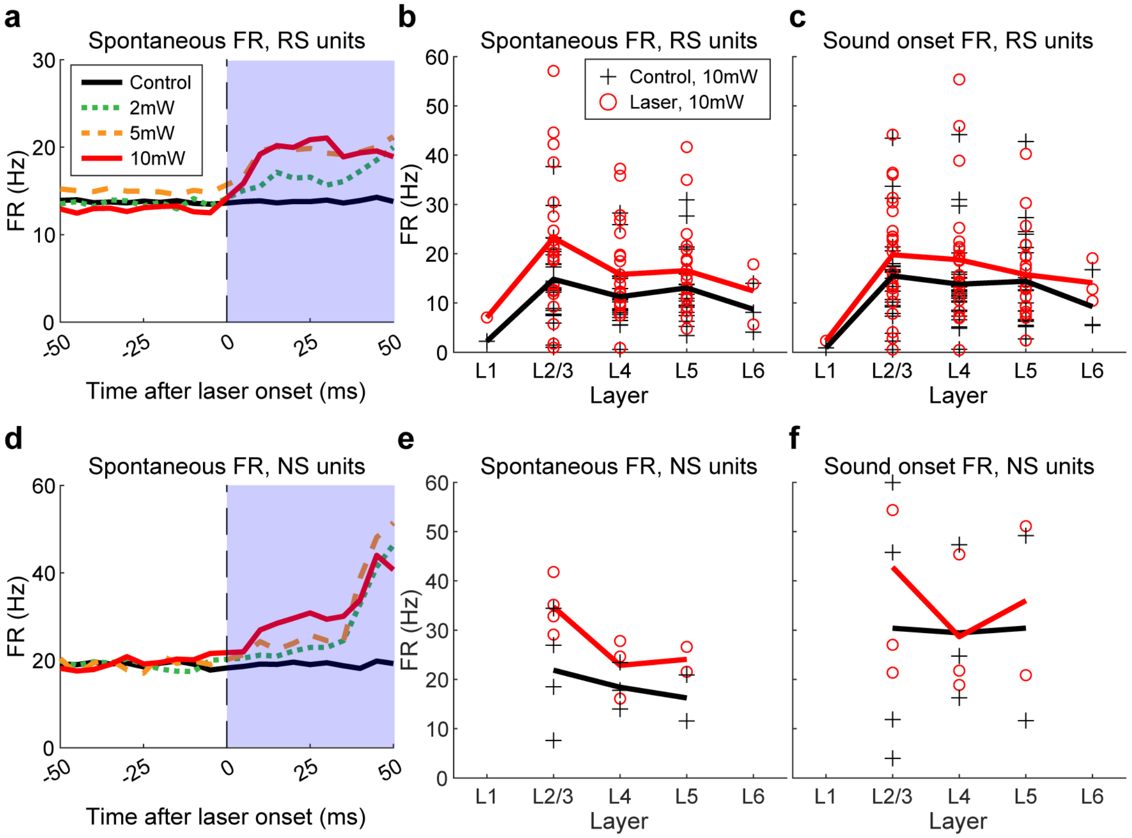

Supplementary Figure 6. Effects of optogenetic suppression on firing rate across layers for regular-spiking (RS) and narrow-spiking (NS) single units. a: Traces of average spontaneous firing rate from 50ms before to 50ms after laser onset (thin dashed line) for control trials (solid black line) and all laser powers (2mW, green dotted line; 5mW orange dashed line; 10mW, red solid line) for all RS units. Blue shaded region represents time period during which laser was on, but no sound was present. During the 100ms shown, no auditory stimulus was presented. The control trace was estimated by pooling all non-optogenetic trials from each of the three blocks. Spontaneous firing rate increases with optogenetic suppression of PV neurons, in a graded manner with laser power, consistent with previous studies. **b:** Spontaneous firing rate for all RS units across layer (L1: n = 1 unit, L2/3: n = 24 units; L4: n = 26 units; L5: n = 22 units; L6: n = 3 units) during control and laser trials at the 10mW recording block, estimated during the 50ms-long blue region in a. L1 was excluded from further analysis due to the small sample size. Repeated-measures ANOVA found laser ($p(3,71) = 6.94e-04$, $\eta^2_p = 0.15$) as a significant factor, but not layer ($p(3,71) = 0.094$, η^2_p = 0.09) or the interaction between the two ($p(3,71) = 0.213$, $\eta^2_p = 0.06$). Within layers, post-hoc tests found highly significant differences between conditions in L2/3 (p = 5.62e-06, d = -0.67) and L4 (p = 0.00760, d = -0.61). **c:** Sound onset firing rate (the first 0.5 s of stimulus presentation across both clean and masked trials) for RS units across layer during the 10mW recording block. Repeated-measures ANOVA yielded laser as a significant factor ($p(3,71) = 5.80e-06$, $\eta^2_p = 0.25$) but not for layer ($p(3,71) = 0.692$, $\eta^2_p = 0.02$) or interaction between the two ($p(3,71) = 0.050$, $\eta^2_p = 0.10$). Within layers, post-hoc tests found highly significant differences between conditions in L2/3 (p = 2.47e-05, d = -0.40) and L4 (p = 6.887e-07, d = -0.47). **d:** Traces of average spontaneous firing rate from 50ms before to 50ms after laser onset (thin dashed line) for control trials (solid black line) and all laser powers (2mW, green dotted line; 5mW orange dashed line; 10mW, red solid line) for all NS units. Counterintuitively, but consistent with the study by Moore et al., spontaneous firing rate for NS units also increases upon PV suppression. **e:** Spontaneous firing rate for all regular-spiking single units across layer (L2/3: n = 4 units; L4: n = 3 units; L5: n = 2 units) during control and laser trials at the 10mW recording block, estimated during the 50ms-long blue region in d. Repeated-measures ANOVA was not calculated due to the small sample size per group. **f:** Sound

onset firing rate for narrow-spiking single units across layer during the 10mW recording block. As with the data in e, repeated-measures ANOVA was not calculated due to the small sample size per group.

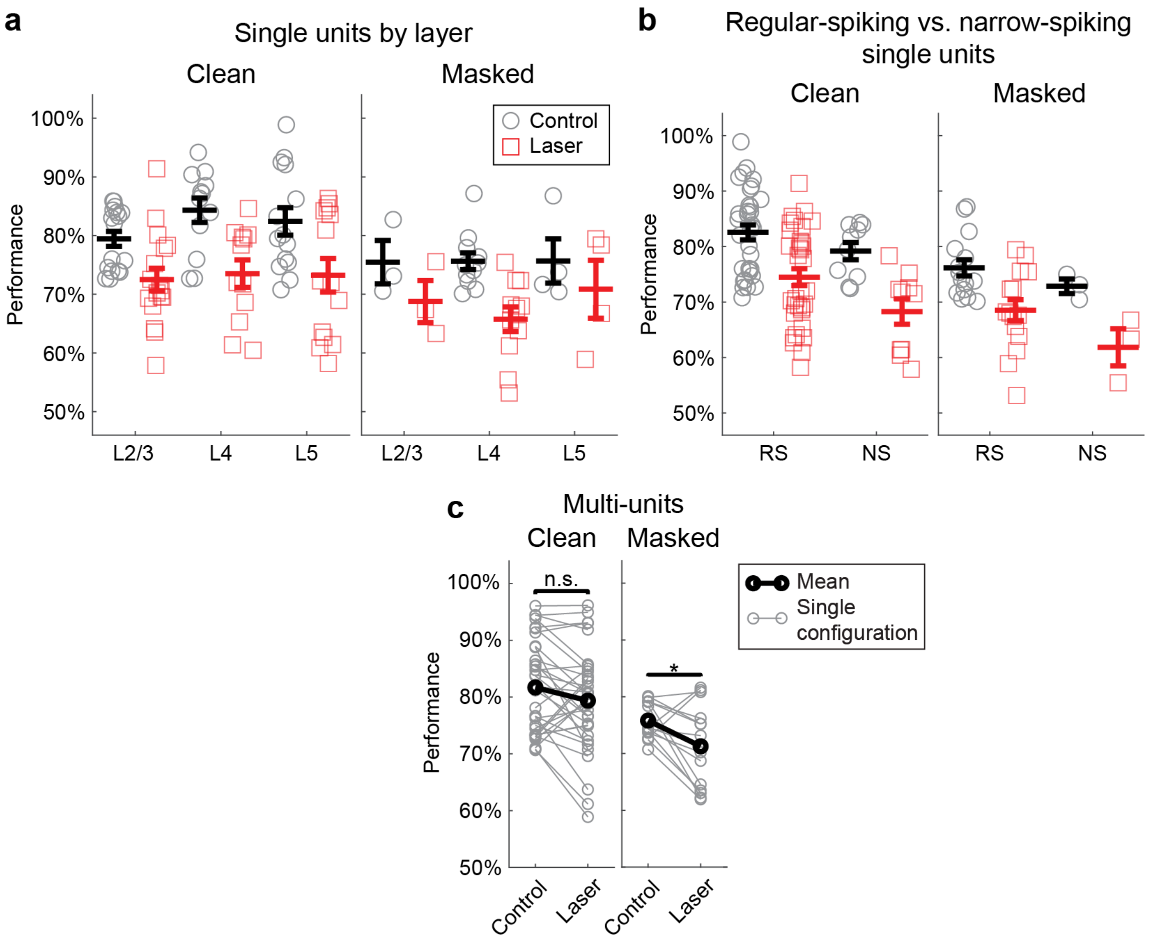

Supplementary Figure 7. Comparison of SPIKE-distance-based performance across different unit types. a: Comparisons between layers for all single units with hotspots (L2/3: *n* = 17 clean configurations, *n* = 3 masked configurations; L4: *n* = 12 clean configurations, *n* = 11 masked configurations; L5: *n* = 14 clean configurations, *n* = 4 masked configurations), with error bars representing SEM. Repeated-measures ANOVA for clean trials yielded significance for laser ($p(2,40) = 3.09\text{e-}10$, $\eta^2_p = 0.63$) but not for layer ($p(2,40) = 0.549$, $\eta^2_p = 0.03$) or interaction ($p(2,40) = 0.338$, $\eta^2_p = 0.05$). Masked ANOVA also yielded significance for laser ($p(2,15) = 0.00412$, $\eta^2_p = 0.43$) but not for layer ($p(2,15) = 0.710$, $\eta^2_p = 0.04$) or interaction ($p(2,15) = 0.508$, $\eta^2_p = 0.09$). **b:** Comparisons between regular-spiking (RS: *n* = 33 clean configurations, *n* = 15 masked configurations) and narrow-spiking (NS: *n* = 10 clean configurations, *n* = 3 masked configurations) single units. Repeated-measures ANOVA for clean trials yielded a significant effect from laser ($p(1,41) = 3.26\text{e-}09$, $\eta^2_p = 0.58$) but not for unit type ($p(1,41) = 0.0665$, $\eta^2_p = 0.08$) or the interaction between the two ($p(1,41) = 0.273$, $\eta^2_p = 0.03$). Repeated-measures ANOVA for masked trials yielded laser as a significant factor ($p(1,16) = 0.00144$, $\eta^2_p = 0.48$) but not for unit type ($p(1,16) = 0.135$, $\eta^2_p = 0.13$) or interaction ($p(1,16) = 0.501$, $\eta^2_p = 0.03$). **c:** Comparisons between conditions for multi-units (MUs). MUs showed similar trends to SUs with a decrease in performance upon PV suppression. Paired t-tests did not yield a significant decrease in performance for clean trials ($n = 33$ configurations, $p = 0.0576$, $d = 0.34$) but yielded significance for masked trials ($n = 15$ configurations, $p = 0.0234$, $d = 0.66$).

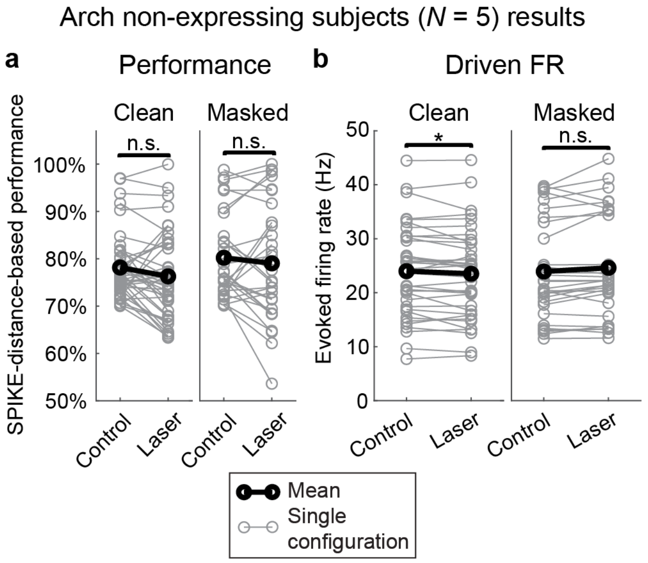

Supplementary Figure 8. Single-units from PV-Cre -non-Arch expressing mice do not show similar changes in discriminability and spiking as PV-Arch-expressing mice. a: Paired comparisons of SPIKE-distance-based performance from control and laser-on trials in n = 5 PV non-Arch expressing mice and n = 19 single units. Paired t-tests did not yield a significant decrease in performance for either clean (n = 40 configurations; p = 0.0734, d = 0.29) or masked (n = 30 configurations; p = 0.4052, d = 0.15) trials when the laser was turned on, which suggests that non-optogenetic effects from the laser did not result in the significant and strong decreases in performance (Figure 3f, Clean: p = 3.44e-10, d = 1.24; Masked: p = 2.50e-04, d = 1.09) seen in our set of PV-Arch-expressing mice. **b:** Paired comparisons of mean evoked firing rate during control and laser trials. Paired t-tests yielded a significant change in spiking during clean trials (p = 0.0386, d = 0.34) but not during masked trials (p = 0.3385, d = -0.35). In addition, we compared the differences in performance and firing rate between conditions (control minus laser) between Arch-expressing and non-Arch-expressing subjects. For performance, an un-paired, 2-sample t-test yielded a significant difference between the two groups in both clean (p = 1.27e-05, d = -1.02) and masked (p = 0.0032, d = -0.93) trials, which indicates that the decrease in performance was larger in Arch-expressing subjects. An un-paired, 2-sample t-test on the change in firing rate during laser condition also yielded a significant difference between the two groups in clean (p = 2.88e-08, d = 1.35) and masked (p = 0.0081, d = 0.82) trials, which indicates that the increase in firing rate was higher in Arch-expressing subjects.

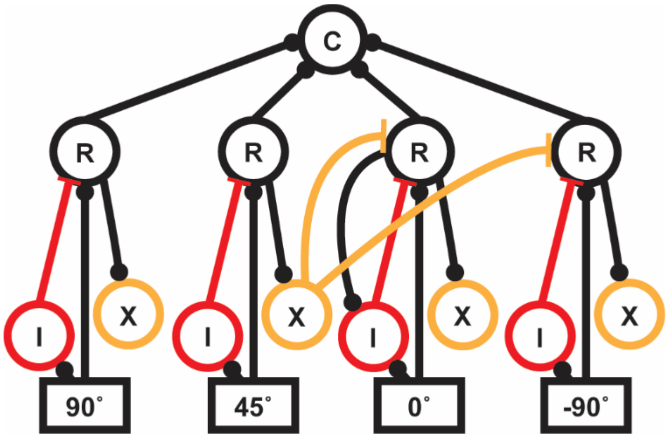

Supplementary Figure 9. Cortical circuits for complex scene analysis. Hypothesized conceptual model of cortical circuit underlying spatial grids. C and R cells represent excitatory units, I cells mediate within-channel inhibition, and X cells mediate cross-channel inhibition.

Supplementary References

1. Allen Brain Reference Atlas - Adult Mouse. Allen Institute for Brain Science (2011). atlas.brain-map.org.
