# Supplementary material for: Parvalbumin neurons enhance temporal coding and reduce cortical noise in complex auditory scenes
Source: Commun Biol. 2023 Jul 19;6:751. doi: 10.1038/s42003-023-05126-0 (PMC10356822; doi:10.1038/s42003-023-05126-0)
Supplement: Supplementary file 3 — Description of Additional Supplementary Files [file 42003_2023_5126_MOESM3_ESM.pdf]

Description of Additional Supplementary Files

File name: Supplementary Data 1

Description: The source data behind the graphs in the paper. Note: Figures 4d and 7b are summary figures of other plots within the paper.
