# Supplementary material for: Parvalbumin neurons enhance temporal coding and reduce cortical noise in complex auditory scenes
Source: Commun Biol. 2023 Jul 19;6:751. doi: 10.1038/s42003-023-05126-0 (PMC10356822; doi:10.1038/s42003-023-05126-0)
Supplement: Supplementary file 5 — Reporting Summary [file 42003_2023_5126_MOESM5_ESM.pdf]

nature portfolio

Corresponding author(s): Sen, COMMSBIO-22-1866B

Last updated by author(s): Jun 23, 2023

Reporting Summary

Nature Portfolio wishes to improve the reproducibility of the work that we publish. This form provides structure for consistency and transparency in reporting. For further information on Nature Portfolio policies, see our Editorial Policies and the Editorial Policy Checklist.

Statistics

For all statistical analyses, confirm that the following items are present in the figure legend, table legend, main text, or Methods section.

n/a	Confirmed	
☐	☒	The exact sample size (*n*) for each experimental group/condition, given as a discrete number and unit of measurement
☐	☒	A statement on whether measurements were taken from distinct samples or whether the same sample was measured repeatedly
☐	☒	The statistical test(s) used AND whether they are one- or two-sided<br>*Only common tests should be described solely by name; describe more complex techniques in the Methods section.*
☐	☒	A description of all covariates tested
☐	☒	A description of any assumptions or corrections, such as tests of normality and adjustment for multiple comparisons
☐	☒	A full description of the statistical parameters including central tendency (e.g. means) or other basic estimates (e.g. regression coefficient) AND variation (e.g. standard deviation) or associated estimates of uncertainty (e.g. confidence intervals)
☐	☒	For null hypothesis testing, the test statistic (e.g. *F*, *t*, *r*) with confidence intervals, effect sizes, degrees of freedom and *P* value noted<br>*Give P values as exact values whenever suitable.*
☒	☐	For Bayesian analysis, information on the choice of priors and Markov chain Monte Carlo settings
☒	☐	For hierarchical and complex designs, identification of the appropriate level for tests and full reporting of outcomes
☐	☒	Estimates of effect sizes (e.g. Cohen's *d*, Pearson's *r*), indicating how they were calculated

Our web collection on statistics for biologists contains articles on many of the points above.

Software and code

Policy information about availability of computer code

Data collection	Data was collected using the Synapse software from Tucker-Davis Technologies (TDT) and custom-made DSP circuits on TDT's Real-Time Processor Visual Design Studio.
Data analysis	For spike sorting, the third-party MATLAB package Kilosort2 was used. To manually curate spike clusters from Kilosort, the Python-based phy2 toolbox and the third-party MATLAB-based spikes toolbox were used. For additional sorting of single-unit clusters, the third-party MATLAB-based sortingQuality toolbox was used. After spike sorting, custom-made MATLAB code was used to carry out the remainder of the analysis.

For manuscripts utilizing custom algorithms or software that are central to the research but not yet described in published literature, software must be made available to editors and reviewers. We strongly encourage code deposition in a community repository (e.g. GitHub). See the Nature Portfolio guidelines for submitting code & software for further information.

Data

Policy information about availability of data

All manuscripts must include a data availability statement. This statement should provide the following information, where applicable:

- Accession codes, unique identifiers, or web links for publicly available datasets
- A description of any restrictions on data availability
- For clinical datasets or third party data, please ensure that the statement adheres to our policy

Source data for the figures are provided in Supplemental Data 1. The data used in this study are available upon reasonable request.

Human research participants

Policy information about studies involving human research participants and Sex and Gender in Research.

Reporting on sex and gender	This study did not involve the use of human participants.
Population characteristics	This study did not involve the use of human participants.
Recruitment	This study did not involve the use of human participants.
Ethics oversight	This study did not involve the use of human participants.

Note that full information on the approval of the study protocol must also be provided in the manuscript.

Field-specific reporting

Please select the one below that is the best fit for your research. If you are not sure, read the appropriate sections before making your selection.

☒ Life sciences ☐ Behavioural & social sciences ☐ Ecological, evolutionary & environmental sciences

For a reference copy of the document with all sections, see nature.com/documents/nr-reporting-summary-flat.pdf

Life sciences study design

All studies must disclose on these points even when the disclosure is negative.

Sample size	Sample sizes are based on norms within the field and were estimated based on neuron yields from our previous publications and pilot studies. A power analysis was performed using G*Power 3.1.9.2. We applied a two-tailed Wilcoxon signed rank test utilizing an β/α ratio=1.0, an effect size of 0.8. Additional assumptions included the expectations of 6-12 neurons/animal. Applying these assumptions resulted in α and β probabilities of <0.1 with 8 subjects.
Data exclusions	Certain units were excluded from analysis if the spiking responses showed a high degree of artifact even after automated removal. Responses at certain spatial grid configurations from a given unit were excluded from analysis if at least 3 of the 10 trials per target identity showed zero spiking, as the reduced amount of responses would result in a coarser calculation of the template-based neural discrimination performance.
Replication	All experimental recordings for a given subject were done in successive blocks of increasing opto-genetic laser power (2mW, 5mW, and 10mW) within a single day.
Randomization	Subjects were not randomly allocated during this study, as all subjects in this study had the same genotype.
Blinding	Investigators were not blinded during data collection or analysis. Blinding was not necessary, as both experimental recordings and data analysis procedures were consistent across all subjects in this study.

Reporting for specific materials, systems and methods

We require information from authors about some types of materials, experimental systems and methods used in many studies. Here, indicate whether each material, system or method listed is relevant to your study. If you are not sure if a list item applies to your research, read the appropriate section before selecting a response.

Materials & experimental systems

n/a	Involved in the study	
☒	☐	Antibodies
☒	☐	Eukaryotic cell lines
☒	☐	Palaeontology and archaeology
☐	☒	Animals and other organisms
☒	☐	Clinical data
☒	☐	Dual use research of concern

Methods

n/a	Involved in the study	
☒	☐	ChIP-seq
☒	☐	Flow cytometry
☒	☐	MRI-based neuroimaging

Animals and other research organisms

Policy information about studies involving animals; ARRIVE guidelines recommended for reporting animal research, and Sex and Gender in Research

Laboratory animals	A total of 14 C57BL6/J transgenic mice were used in this study. Original breeding pairs of parvalbumin-Cre (PV-Cre: B6;129P2-Pvalbtm1(cre)Arbr/J), and Ai40 mice (Arch: B6.Cg-Gt(ROSA)26Sortm40.1(CAG-aop3/EGFP)Hze/J) mice were obtained from Jackson Laboratory (Maine), and all breeding was done in house. Subjects consisted of both male and female PV-Arch offspring 8-12 weeks old on the day of recording.
Wild animals	The study did not involve the use of wild animals.
Reporting on sex	Findings in this study apply to both male and female mice. Animal sex was not considered in study design.
Field-collected samples	The study did not involve the use of field-collected samples.
Ethics oversight	All procedures involving animals were approved by the Boston University Institutional Animal Care and Use Committee and the University of Illinois at Urbana-Champaign Institutional Animal Care and Use Committee (IACUC).

Note that full information on the approval of the study protocol must also be provided in the manuscript.
